# Comparative Study of 0.2% Glyceryl Trinitrate Ointment for Pain Reduction after Hemorrhoidectomy Surgery

**DOI:** 10.1055/s-0039-3400532

**Published:** 2019-12-02

**Authors:** Sepideh Vahabi, Siavash Beiranvand, Arash Karimi, Mahmoudreza Moradkhani

**Affiliations:** 1 Department of Anesthesiology, Faculty of Medicine, Lorestan University of Medical Sciences, Khorramabad, Iran

**Keywords:** glyceryl trinitrate ointment, hemorrhoidectomy, VAS

## Abstract

**Context** Hemorrhoid is one of the most common diseases in both, men and women, affecting half of the world's population over the age of 50.

**Aims** The aim of this study was to evaluate the analgesic effects of local ointment of glyceryl trinitrate ointment (GTN) after hemorrhoidectomy.

**Methods and Materials** In this randomized double-blind, placebo-controlled study, the patients were grouped as the treatment, that is GTN, and placebo (P) group. After surgery, 0.2% gelatin GTN ointment (250 mg), and P ointment ( *n* = 20 for each group) were applied topically on 1 cm on the anus using a standard ruler, three times a week in respective groups. visual analog scale was used to assess the intensity of the pain and complications of the drugs were observed at 6, 12, 18, and 24 hours.

**Statistical Analysis Used** Data and questionnaires were analyzed statistically using SPSS17 software and results were recorded in the tabular form.

**Results** Six hours after the application of the ointment, no significant difference was found among the groups, however, after 12, 18, and 24 hours significant reduction in pain was seen in GTN group, which was least after 18 hours. The mean values of the total pain score in the first 24 hours after surgery in the GTN group were 3.15 and 5.45 in the P group which were statistically significant. Nonetheless, headache was significantly increased in the GTN group.

**Conclusion** Simple and safe topical GTN ointment can reduce the pain after hemorrhoidectomy, leading to the reduced need of other analgesics.

Hemorrhoid is one of the most common diseases in both, men and women, affecting half of the world's population over the age of 50. 1 Hemorrhoids are graded according to the grade of prolapse, where, in patients with III and IV degrees, hemorrhoidectomy is required. 1 Surgery is one of the main procedures used to provide timely treatment particularly in patients where conventional methods have failed, leading to the development of the complications. Of the various surgical procedures, the Milligan-Morgan hemorrhoidectomy is regarded as the “gold standard” treatment for hemorrhoids. 2 3 Nonetheless, encountering postoperative pain is one of the major challenges. Studies have reported hemorrhoidectomy as a very painful procedure involving the need for the opioids in pain management, in 20 to 40% of the patients. 4

Poorly managed postoperative pain can result in prolonged rehabilitation and complications in patients. 5 Conventionally, opioids and NSAIDs are chiefly exploited for postoperative pain management, however, drug dependence has threatened their use. Pain relief can lead to a quicker recovery and reduced anxiety in the patient. 6 An internal sphincter plays an important role in hemorrhoidal diseases and is regarded as the source of pain after a hemorrhoid operation. 7

In recent years, vasodilators such as glyceryl trinitrate ointment (GTN), by relaxing the smooth muscles of the canal, have reduced spasm of the sphincter, causing chemical sphincterotomy to be reversible. 8 This property is used for the treatment of chronic anal fissure, and up to 70% improvement has been reported. Thus, the complications of sphincterotomy surgery, such as incidence of incontinence, are prevented. 9 It has been proven that sphincterotomy reduces pain after hemorrhoidectomy. 6 8 Topical use of GTN ointment can reduce internal pressure and anal pain. 10 Such topical ointment can reduce the need of analgesics and associated adverse effect. Therefore, we designed this study to evaluate the analgesic effects of local ointment of GTN in the anus for pain management after hemorrhoidectomy.

## Materials and Methods

This double-blind, placebo-controlled prospective randomized clinical trial, was conducted in Shohada Ashayer hospital, Khorramabad in 2017.

The study population included patients aged 18 to 60 years in American Society of Anesthesiologists class I and II. Exclusion criteria included the presence of cardiovascular disease, pregnancy, history of headache and migraine, hypotension, anaphylaxis, dermatitis, coagulation and drug dependence, and the recent use of GTN in any form of the drug. Patients' information such as name, case number, age, gender, previous use of drugs, history of addiction, any type of bleeding, anal pain, other diseases, chronic headache, dermatitis, low blood pressure, and duration of hemorrhoids were included in the questionnaire.

The surgeon identified the degree of hemorrhoids and its accompaniment.

Patients who were eligible to enter into the study were randomly assigned to the treatment and placebo groups according to a computer-generated list. At the time of the trial, the randomization code was not available by the investigators. The code was broken 2 months after the end of the first trial. During the time of follow-up (first and second postoperative months), an examiner who was unaware of the course of the treatment, assessed the outcomes.

In this double-blind, placebo-controlled prospective randomized clinical trial, Milligan-Morgan hemorrhoidectomy was performed under spinal anesthesia with 2 cm ^3^ 5% lidocaine using L2-L3 or L4 needle. The L3 was fitted with a horse saddle block. 0.2% GTN was prepared at the hospital drug store by the pharmacist by achieving the dilution of commercial GTN with paraffin.

Relaxation of the internal anal sphincter is mediated by the release of nitric acid by the nerves. 7 11 12 Local GTN can act as a nitric oxide feeder 13 and has similar effects in reducing the pressure of the rectal canal.

At the end of the surgery, patients were randomly divided into two groups according to the random numbers table. The first group GTN ( *n* = 20) was administered with 0.2% gelatin GTN (250 mg) ointment and group P ( *n* = 20) was provided and the patients were instructed to apply the provided ointment in the anal canal using a cotton swab. After the surgery, patients were initially transferred to the recovery unit. Following recovery, in the surgical ward, patients were provided with the ointment to be applied on a centimeter of the anus using a standard ruler, three times a day.

The first dose was given after surgery in the operating room and two other doses at 8-hour intervals in the surgical ward. The pain score was evaluated using visual analog scale (VAS) based on the patient's report, with pain intensity ranging from 0 to 10, where 0 represents the absence of the pain and 10 is the greatest intensity of the pain felt by the patient. In case of VAS greater than 5, patients were prescribed with 1 mg/kg of intramuscular meperidine. Majag syrup was given to all the patients to aid bowel movements.

After operation for 6, 12, 18, and 24 hours, vital signs and adverse effects such as headache, local dermatitis, hypertension, and more than 20% preoperative bleeding from the wound site were recorded (based on grading from 0 to 3) whereas, the frequency of receiving meperidine was recorded in the questionnaire.

Postoperative wound site bleeding was evaluated based on the amount of blood in the dressing gauze from 0 to 3.

Grade 0: Gauze is not bloody.
Grade 1: A few blood spots.
Grade 2: The gauze of the dressing area is completely bloody.
Grade 3: Blood from the site of the dressing area.

The headache rating in this study was from zero to three:

Grade 0: No headache.
Grade 1: Mild headache.
Grade 2: Moderate headache.
Grade 3: Severe headache.

A written consent form was obtained from all the patients enrolled in this study. The study was approved by The Research Ethics Committee of Lorestan University of Medical Sciences, Khorramabad, Iran.

After collecting data, questionnaires were analyzed statistically using SPSS17 software and results were recorded in the tabular form. To describe and compare the mean values from *t* -student and chi-square, and to compare them with nondominant variables, Fisher's exact test was used. Nineteen patients in each group were used to calculate sample size and to obtain a power of 90% for this study. *p* -Value <0.05 was considered to be significant.

## Results

Analysis of demographic characteristics by T-independent tests showed that there was no significant difference between the mean age of the two groups, that is, the age was homogeneous among the groups (DF = 148, *p* = 0.69, *t* = −0.4). Chi-square test showed that there was no significant relationship between sex and the groups studied ( *p* = 1, degrees of freedom [DF] = 1, *X* ^2^ = 0). Similarly, independent *t* -test indicated that there was no significant difference between the distribution of hemorrhoids in the groups (H = 0.33). Although the mean of the degree of pain 6 hours after the surgery was not significantly different between both the groups, however, after 12, 18, and 24 hours the pain in GTN group was significantly reduced, which was least after 18 hours ( Table 1 ).

**Table 1:** Comparison of the mean pain intensity based on the visual acuity scale of 12, 18, 24, and 6 h after ointment in both groups using GTN and placebo

**Severity of pain in 6 h later** **Group**	**Average**	**Standard deviation**	**Minimum**	**Maximum**	**Number**
GTN	5.1	1.5	1	7	20
Placebo	5.3	1.3	1	7	20
Total	3.3	1.4	1	7	40
*t* = −1.66, *p* = 0.99, DF = 148.					
**Severity of pain in 12 h later** **Group**	**Average**	**Standard deviation**	**Minimum**	**Maximum**	**Number**
GTN	3.4	1.6	0	5–6	20
Placebo	5.2	1.6	0	7–8	20
Total	3.4	1.6	0	9	40
*p* = 0.001, DF = 148, *t* = −1.43.					
**Severity of pain in 18 h later** **Group**	**Average**	**Standard deviation**	**Minimum**	**Maximum**	**Number**
GTN	2	1.5	1	7	20
Placebo	5	1.3	1	7	20
Total	2.2	1.4	1	7	40
*p* < 0.005, DF = 148, *t* = −1.66					
**Severity of pain in the next 24 h** **Group**	**Average**	**Standard deviation**	**Minimum**	**Maximum**	**Number**
GTN	2.1	1.9	2	10	20
Placebo	6.3	1.4	3	9	20
Total	3.7	1.7	2	10	40
*p* = 0.001, DF = 137.6, *t* = −1.38					

Abbreviation: GTN, glyceryl trinitrate ointment.

The mean values of the total pain score in the first 24 hours after surgery in the GTN and placebo groups were 3.15 and 5.45, respectively where these differences were statistically significant (p < 0.005). For patients with a degree of pain greater than 5, 1 mg/Kg intramuscular meperidine was administered, where the need of meperidine was lower in GTN group compared with the placebo ( *p* = 0.001; Table 2 ).

**Table 2:** Frequency distribution of meperidine received in the first 24 h after surgery in the two groups

Number of receiving meperidine	Once	Twice	Three times or more
Group GTN	4 people	1 person	0 person
Placebo group	3 people	10 people	1 person
Total	7	11	1
*p* = 0, DF = 143, *t* = −1.22			

Abbreviation: GTN, glyceryl trinitrate ointment.

Bleeding was not reported in either group and this difference was not statistically significant, measured by Chi-square test ( *p* = 0.3038). Dermatitis was seen in a patient in GTN group seen as inflammation and slight redness of the anterior perineal region, however, it was not statistically different between the groups ( *p* = 0.3038).

Three patients in the GTN group and two in the P group were reported to present hypertension, however, the difference was not statistically significant ( *p* = 0.188).

Anaphylaxis was not reported in either group ( *p* = 0.066). However, 12 patients in the GTN group reported having headache (60%), where, two of them had severe headache, five presented moderate and mild headache, respectively. On the other hand, three people in the P group had a mild headache (15%). Statistical analysis showed that headache was not significantly greater in the GTN group ( Table 3 ).

**Table 3:** Frequency distribution of headache in the two groups

	Headache Grade 0	Headache Grade 1	Headache Grade 2	Headache Grade 3	*p* -Value
Group GTN	8	5	5	2	0.066
Group P	17	3	0	0	0.066
Total	25	8	5	2	
	*p* = 0.087, DF = 1, *X* ^2^ = 1.7				

## Discussion

For patients with first-degree hemorrhoids, nonsurgical treatments include diet and oral flavonoids, and topical drugs. Nonetheless, some patients fail to respond to these conventional methods and worsening of the disease requires plastic bandage ligation, sclerotherapy, infrared coagulation, analgesic dilatation by Lords, and hemorrhoidectomy. 10 Vascular relaxant, such as GTN, relaxes the smooth muscles of the canal and reduces the pressure at internal sphincter making chemical sphincterotomy reversible. 8 On the other hand, sphincterotomy surgery can reduce pain after hemorrhoidectomy. 6 8 These findings have led us to consider that GTN can play a similar role in the treatment of postoperative spasticity, which can be one of the causes of postoperative pain.

The internal anal sphincter of patients with hemorrhoids exhibits abnormal rhythm of contraction and greater pressure is required for the contraction of the anus, than the healthy individuals, 14 and the abnormality of the sphincter is associated with hemorrhoid. GTN reduces the maximum relaxation pressure of the anal canal in patients with chronic fractures, 6 8 15 and a significant reduction in the pain has been reported despite the presence of fracture. 15 There is no standard dose for the topical application of GTN. 10 Cavcić et al, 16 demonstrated the effect of GTN on treating thromboembolic hemorrhoids in 2001. 16 In 2005, Silverman et al used calcium channel blockers to treat post hemorrhoidectomy pain. 17

The randomized double-blind placebo-controlled study was conducted by the Wasvary et al 18 in the United States on hemorrhoidectomy patients where 19 patients were treated with 2% GTN ointment for postoperative pain management and placebo study was conducted on 20 patients. Patients were advised to use hydrocodone bitartrate if needed. After the operation, the degree of pain, complications of the drug, and the frequency of receiving the housing in the questionnaire were recorded by the VAS method. It was concluded that the GTN group had a lesser degree of the pain than the placebo group, however, it was not statistically significant (4.35 vs. 4.47 with *p* = 961). Hospitalization was a day higher in the placebo group and increased significantly from day-2 after the surgery ( *p* < 0.05). The complications of using the ointment in the GTN group were significantly higher ( *p* < 0.002) and included eight cases of headache, required nonopioid analgesics. 18 In this study, following a day after the surgery, the mean score of pain was significantly lower in GTN group (3.15 against 5.45) and the need of meperidine was significantly reduced. Conversely, the incidence of postoperative headache in GTN group showed a significant increase (60 vs. 42%).

In contrast to Wasvary et al, 18 our results were restricted to the 24 hours of the postoperative period. Coskun et al 19 reported that the beneficial effects of GTN ointment after hemorrhoidectomy were limited to patients who had a high anal resting pressure before the surgery. 19

A study by Tjandra et al, 10 in Melbourne, Australia, in 2007, showed that GTN ointment improves the hemorrhoid-associated symptoms by 2.9% when the anal canal resting pressure is high. A total of 58 patients presented hemorrhoids of Grades 1 and 2, and maximum relaxation pressure of the anal canal greater than 70 mm Hg. GTN ointment was administered topically twice a day for 14 days and followed for 28 days. The incidence of bleeding, VAS, itching, inflammation, and the intestinal movements were significantly less. But, 43% of the patients suffered from headache, therefore it was concluded that the drug can impose potential side effects on patients with low-level, high-blood pressure. 10 Despite, no surgical procedure was performed in this study, hemorrhoids-related symptoms were significantly reduced by using the GTN ointment. The incidence of headache in this study was lower than our study (43 vs. 60%). The limitation of our study is the low number of sample size therefore, future studies with long-term follow-up and larger sample size are required.

In conclusion, simple and safe use of GNT significantly reduces pain in postoperative hemorrhoidectomy patients and is associated with lesser need of opioids.

## References

[1] Stamos M J Colon and rectal surgery J Am Coll Surg 1998 186 02 134 140 9482615 10.1016/s1072-7515(98)00009-x

[2] Farouk R Duthie G S MacGregor A B Bartolo D CC Sustained internal sphincter hypertonia in patients with chronic anal fissure Dis Colon Rectum 1994 37 05 424 429 8181401 10.1007/BF02076185

[3] American Society of Anesthesiologists Task Force on Acute Pain Management.Practice guidelines for acute pain management in the perioperative setting: an updated report by the American Society of Anesthesiologists Task Force on Acute Pain Management Anesthesiology 2012 116 02 248 273 22227789 10.1097/ALN.0b013e31823c1030

[4] Brennan F Carr D B Cousins M Pain management: a fundamental human right Anesth Analg 2007 105 01 205 221 17578977 10.1213/01.ane.0000268145.52345.55

[5] Kehlet H Holte K Effect of postoperative analgesia on surgical outcome Br J Anaesth 2001 87 01 62 72 11460814 10.1093/bja/87.1.62

[6] Carapeti E A Kamm M A McDonald P J Chadwick S J Melville D Phillips R K Randomised controlled trial shows that glyceryl trinitrate heals anal fissures, higher doses are not more effective, and there is a high recurrence rate Gut 1999 44 05 727 730 10205213 10.1136/gut.44.5.727 PMC1727506

[7] Gorfine S R Treatment of benign anal disease with topical nitroglycerin Dis Colon Rectum 1995 38 05 453 456, discussion 456–457 7736873 10.1007/BF02148842

[8] Lund J N Scholefield J H A randomised, prospective, double-blind, placebo-controlled trial of glyceryl trinitrate ointment in treatment of anal fissure Lancet 1997 349(9044):11 14 8988115 10.1016/S0140-6736(96)06090-4

[9] García-Aguilar J Belmonte Montes C Perez J J Jensen L Madoff R D Wong W D Incontinence after lateral internal sphincterotomy: anatomic and functional evaluation Dis Colon Rectum 1998 41 04 423 427 9559625 10.1007/BF02235754

[10] Tjandra J J Tan J JY Lim J F Murray-Green C Kennedy M L Lubowski D Z Rectogesic (glyceryl trinitrate 0.2%) ointment relieves symptoms of haemorrhoids associated with high resting anal canal pressures Colorectal Dis 2007 9 05 457 463 17504344 10.1111/j.1463-1318.2006.01134.x

[11] Rattan S Sarkar A Chakder S Nitric oxide pathway in rectoanal inhibitory reflex of opossum internal anal sphincter Gastroenterology 1992 103 01 43 50 1612356 10.1016/0016-5085(92)91093-j

[12] Tøttrup A Ny L Alm P Larsson B Forman A Andersson K E The role of the L-arginine/nitric oxide pathway for relaxation of the human lower oesophageal sphincter Acta Physiol Scand 1993 149 04 451 459 8128894 10.1111/j.1748-1716.1993.tb09642.x

[13] Loder P B Kamm M A Nicholls R J Phillips R KS ‘Reversible chemical sphincterotomy’ by local application of glyceryl trinitrate Br J Surg 1994 81 09 1386 1389 7953427 10.1002/bjs.1800810949

[14] Lohsiriwat V Treatment of hemorrhoids: a coloproctologist's view World J Gastroenterol 2015 21 31 9245 9252 26309351 10.3748/wjg.v21.i31.9245 PMC4541377

[15] Kennedy M L Sowter S Nguyen H Lubowski D Z Glyceryl trinitrate ointment for the treatment of chronic anal fissure: results of a placebo-controlled trial and long-term follow-up Dis Colon Rectum 1999 42 08 1000 1006 10458121 10.1007/BF02236691

[16] Cavcić J Turcić J Martinac P Mestrović T Mladina R Pezerović-Panijan R Comparison of topically applied 0.2% glyceryl trinitrate ointment, incision and excision in the treatment of perianal thrombosis Dig Liver Dis 2001 33 04 335 340 11432512 10.1016/s1590-8658(01)80088-8

[17] Silverman R Bendick P J Wasvary H J A randomized, prospective, double-blind, placebo-controlled trial of the effect of a calcium channel blocker ointment on pain after hemorrhoidectomy Dis Colon Rectum 2005 48 10 1913 1916 16175328 10.1007/s10350-005-0135-4

[18] Wasvary H J Hain J Mosed-Vogel M Bendick P Barkel D C Klein S N Randomized, prospective, double-blind, placebo-controlled trial of effect of nitroglycerin ointment on pain after hemorrhoidectomy Dis Colon Rectum 2001 44 08 1069 1073 11535840 10.1007/BF02234622

[19] Coskun A Duzgun S A Uzunkoy A Bozer M Aslan O Canbeyli B Nitroderm TTS band application for pain after hemorrhoidectomy Dis Colon Rectum 2001 44 05 680 685 11357030 10.1007/BF02234566

